# Follicular adenoma with a papillary architecture originating from an ectopic thyroid gland: a case report

**DOI:** 10.1186/s12902-024-01547-y

**Published:** 2024-01-30

**Authors:** Kiyomi Kuba, Tomonori Kawasaki, Yuichiro Enoki, Hitoshi Inoue, Satoko Matsumura, Tomoko Yamazaki, Yasuhiro Ebihara, Mitsuhiko Nakahira, Masashi Sugasawa

**Affiliations:** 1 Department of Head and Neck Surgery, Ageo Central General Hospital, Ageo, Saitama Japan; 2 Department of Diagnostic Pathology, Saitama Medical University International Medical Center, Hidaka, Saitama Japan; 3 Department of Head and Neck Surgery and Otolaryngology, Saitama Medical University International Medical Center, Hidaka, Saitama Japan; 4 Department of Head and Neck Surgery, Kameda Medical Center, Kamogawa, Chiba Japan

**Keywords:** Ectopic thyroid grand, Follicular adenoma, Papillary architecture, Submandibular mass, Thyroid tumor

## Abstract

**Background:**

Follicular adenomas with papillary architecture are rare tumors of thyroid origin and are composed of completely encapsulated follicular cells with a papillary architecture lacking the nuclear characteristics of papillary carcinoma. Herein, we present a case of follicular adenoma with papillary architecture originating from an ectopic thyroid gland, diagnosed from a mass in the submandibular region.

**Case Presentation:**

A 70-year-old woman was referred to our hospital with the chief complaint of a painless left submandibular mass that had been present for one year. The patient underwent left submandibular dissection for therapy and diagnosis. Microscopically, papillary lesions with fibrovascular cores were observed in the interior, and the epithelial cells were cylindrical in shape with eosinophilic cytoplasm, round or oval nuclei, with no pathological features, leading to a diagnosis of papillary carcinoma or follicular carcinoma. The mass was diagnosed as a follicular thyroid adenoma with papillary architecture. This is the first report of a follicular adenoma with a papillary architecture originating from an ectopic thyroid gland.

**Conclusion:**

This experience suggests that follicular adenoma should be included in the differential diagnosis of ectopic thyroid tumors.

## Introduction

Ectopic thyroid disease is caused by developmental abnormalities during the embryonic period, or seeding from a prior thyroid surgery. The frequency of ectopic thyroid due to embryonic abnormalities is low, ranging from 1 in 100,000 to 300,000 [1], and ectopic thyroids are predominantly located at the midline of the neck, such as the root of the tongue, anterior larynx, or back of the sternum, with presentation on the lateral neck rare [2]. Follicular adenomas with papillary architecture account for 3% of follicular adenomas, and are tumors of thyroid origin composed of completely encapsulated follicular cells. These tumors have a papillary architecture that lacks the nuclear characteristics of papillary carcinoma [3]. In the present study, we describe a case of follicular adenoma with papillary architecture originating from an ectopic thyroid gland, diagnosed from a mass in the submandibular region. This is the first report of a follicular adenoma with papillary architecture originating from an ectopic thyroid.

## Case presentation

**Fig. 1:**
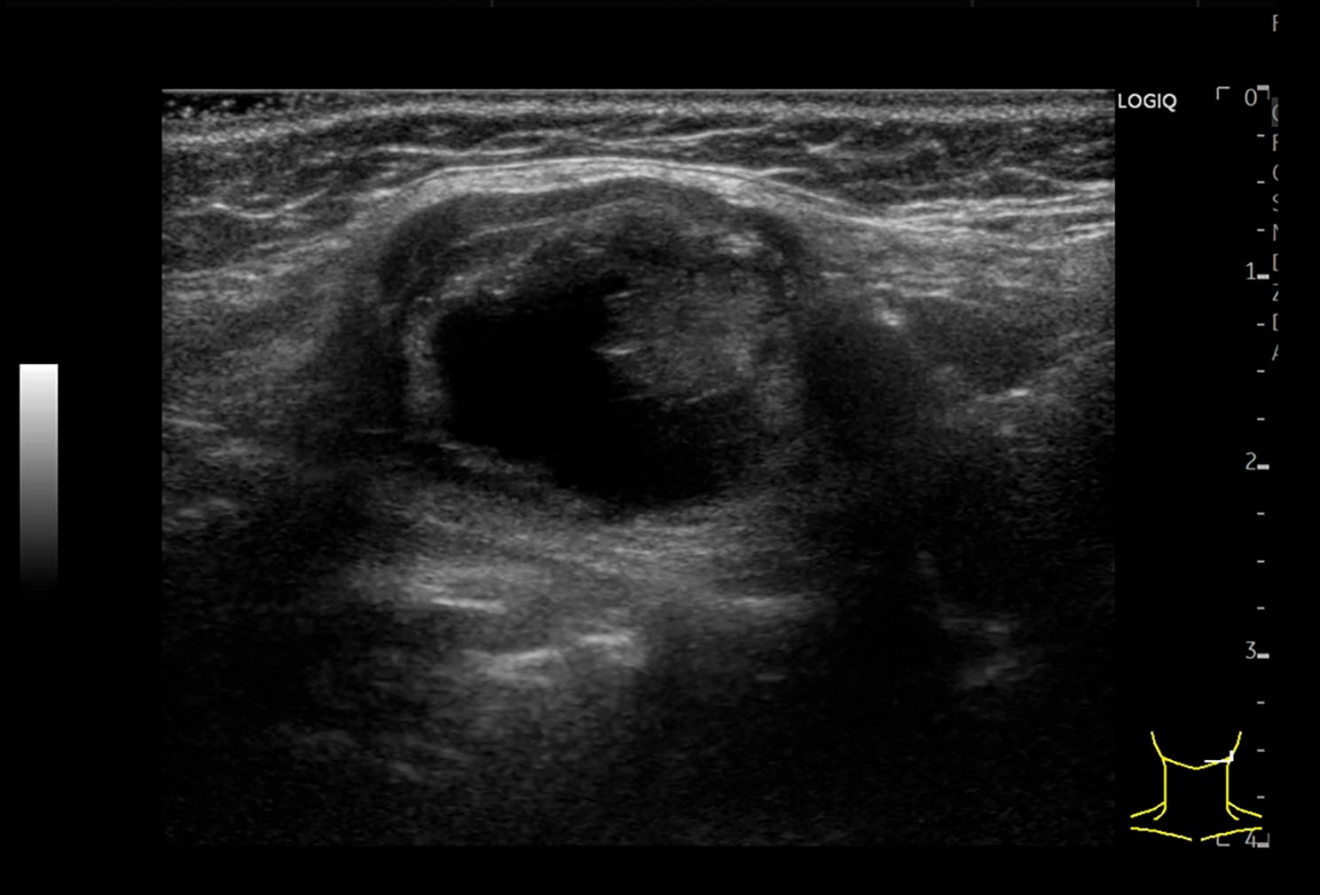
Ultrasonographic images of the tumor. The tumor was located in the left submental region and had a low internal density with mixed papillary hyperechogenicity at the internal margin

**Fig. 2:**
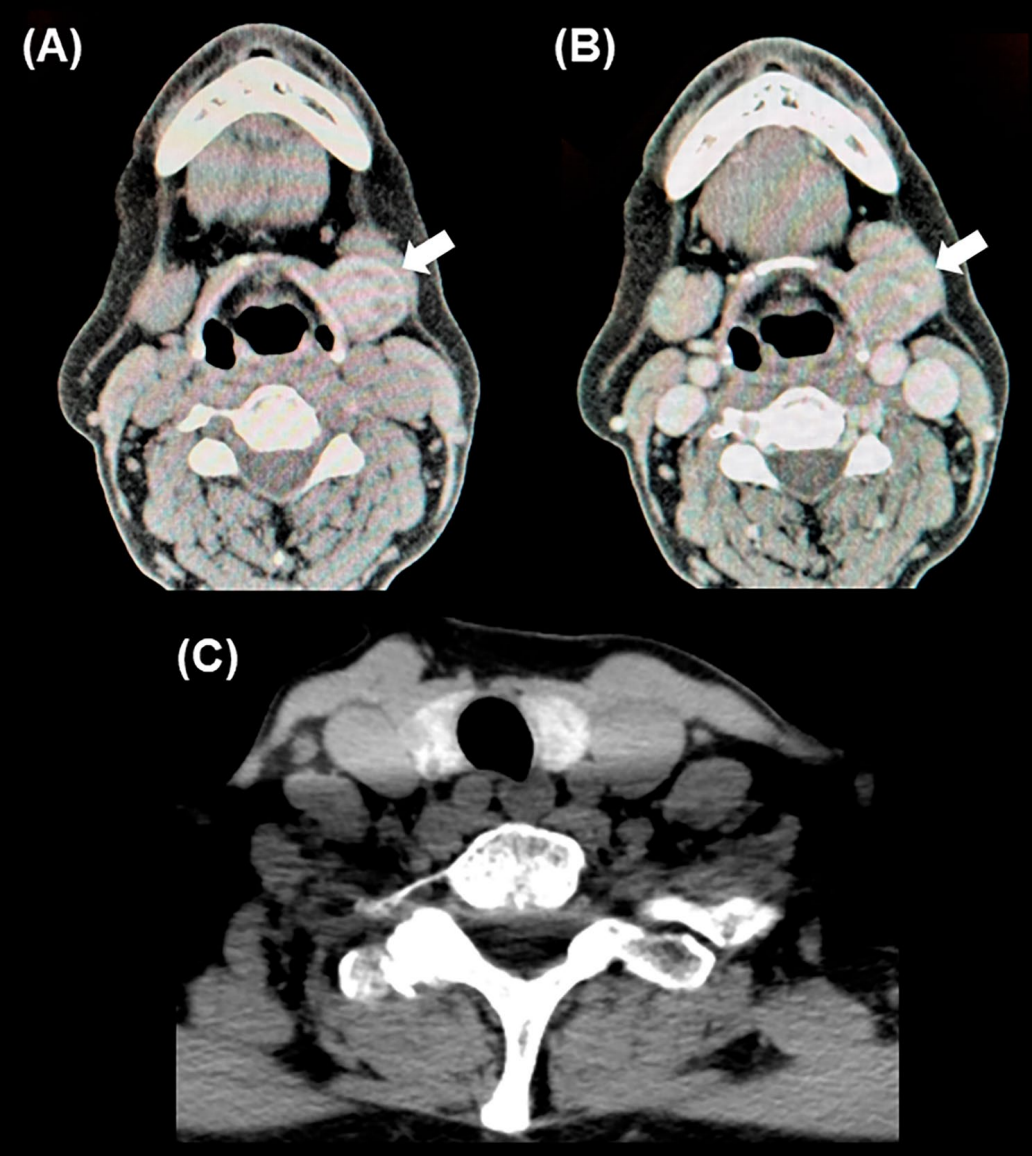
Computed tomography image of the neck. **A** Well-defined mass is located behind the left submandibular gland (white arrow). Without iodine contrast, the mass was mostly hyperdense and had low density. **B** The mass was hardly enhanced by the iodine contrast. **C** Thyroid gland position

A 70-year-old woman was referred to our hospital with the chief complaint of a painless left submandibular mass that had been present for one year. She had no medical conditions other than medicine prescribed for dyslipidemia, and had no previous surgical history. Further, she had never smoked or had no history of alcohol abuse. The mass was approximately 20 mm, with slightly poor mobility and no tenderness, and was suspected to be a submandibular gland tumor. Neck ultrasonography revealed a heterogeneous mass on the dorsal aspect of the left submandibular gland measuring 21 × 20 × 16 mm^3^ with smooth margins, low internal density, and mixed papillary hyperechogenicity at the internal margins (Fig. 1). Malignant lymphoma or metastatic lymph nodes were initially considered as differential diagnoses. The left submandibular gland was normal, the thyroid gland was in a normal position, and multiple 10–20 mm cysts and hypoechoic nodules were observed, suggesting an adenomatous goiter. Cervical computed tomography (CT) revealed a well-defined mass behind the left submandibular gland. Without iodine contrast, the mass was mostly hyperdense, and had partially low density, which was hardly enhanced by iodine contrast, suggestive of lymph node disease (Fig. 2A, B). The thyroid gland was in a normal position (Fig. 2C). Fine-needle aspiration cytology (FNAC) of the left submandibular mass revealed no epithelial cells, and findings of foamy cells on a bloody background were suggestive of cystic fluid, which did not lead to any diagnosis.

**Fig. 3:**
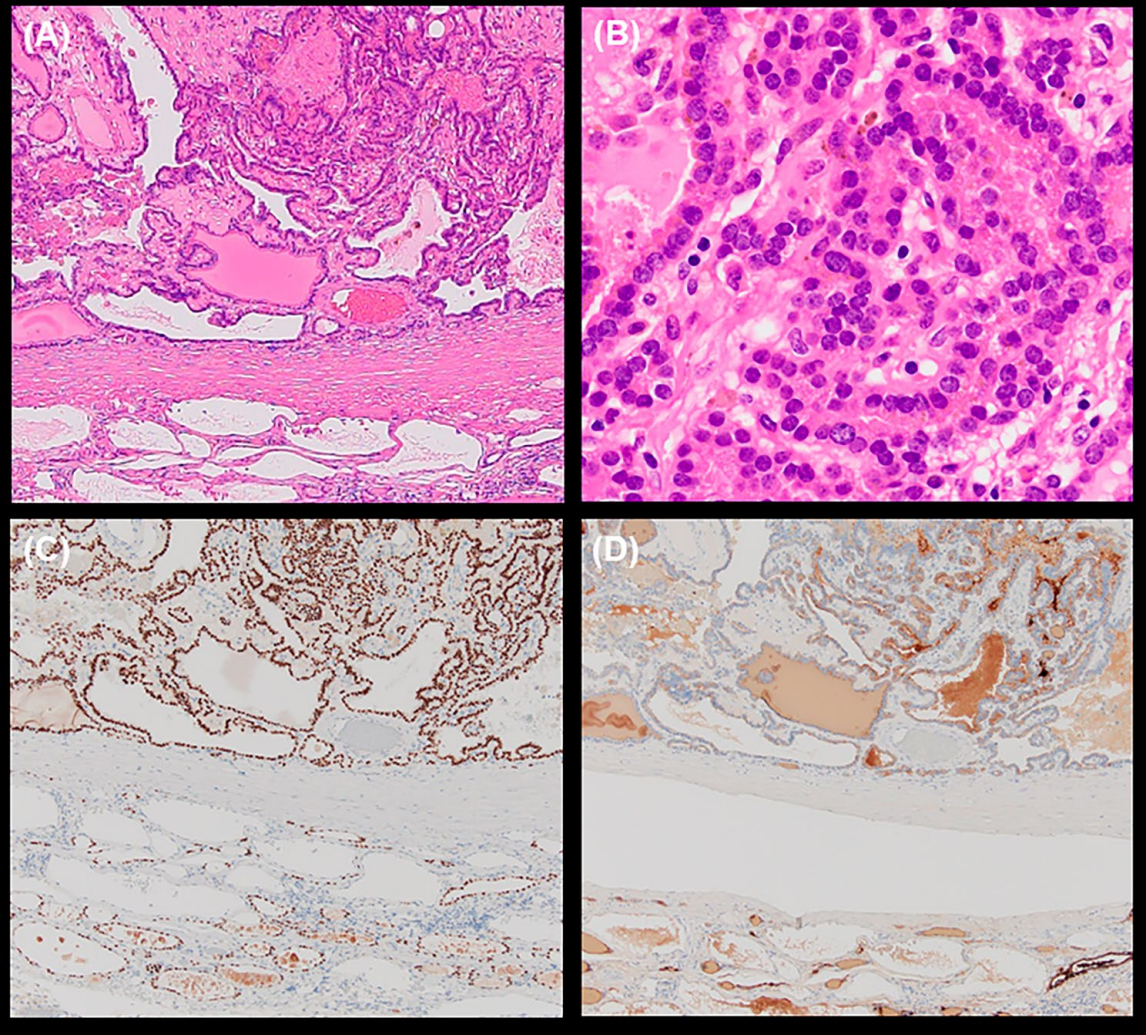
Histopathological examination of the tumor. **A** Papillary lesions with fibrovascular cores are observed in the interior (H&E staining, original magnification×40). **B** Epithelial cells are cylindrical in shape, with eosinophilic cytoplasm and round or oval nuclei (H&E*, original magnification×400). **C** Epithelial cells were positive for TTF-1(original magnification×40). **D** Epithelial cells are positive for thyroglobulin (original magnification×40). *Hematoxylin and Eosin

The patient underwent left submandibular dissection for diagnostic and therapeutic purposes. Intraoperative findings revealed a solitary mass that was not contiguous with either the submandibular or thyroid glands. Histopathological examination revealed a cystic lesion filled with a dark brown jelly-like substance at the macroscopic level. The cyst wall was adherent to the submandibular gland, but without continuity. Microscopically, papillary lesions with fibrovascular cores were observed in the interior, and the epithelial cells were cylindrical in shape, with eosinophilic cytoplasm and round or oval nuclei. Although nuclear grooves were observed in some areas, no intranuclear inclusion bodies or psammoma bodies were detected. The entire lesion was evaluated and no pathological features leading to the diagnosis of papillary carcinoma, and no findings, such as capsular or vascular invasion, to indicate malignancy or follicular carcinoma. Immunohistochemically, the epithelial cells were positive for TTF-1 and thyroglobulin (Fig. 3). Based on these pathological findings, the lesion was diagnosed as a follicular thyroid adenoma with papillary architecture arising from the ectopic thyroid gland. Since the left submandibular mass was a thyroid tumor, FNAC of the left thyroid mass was performed to exclude papillary carcinoma. The results showed sheet-like follicular epithelial cells on a colloidal background. The cellular findings were consistent with those of adenomatous goiter, and papillary carcinoma was ruled out. The postoperative thyroid function was normal, and the patient did not require thyroid replacement therapy. The patient has not experienced any recurrence within two years.

## Discussion

During the embryonic period, the central and lateral anlage of the thyroid gland fuse and eventually descend to their normal position. Ectopic thyroid gland occurs when this normal descent fails. Most cases of ectopic thyroid gland due to developmental anomalies arising along the midline, including the root of the tongue or the anterior neck, and rarely on the lateral side of the neck [38]. Since the first report of an ectopic thyroid in the submental region by Helidonis et al. in 1980, to the best of our knowledge, only 48 papers have been reported to date. These included one report of three cases and one report of two cases, while the rest were single case reports [2, 4–50]. Herein, we provide a brief review these previously-published cases. The age at diagnosis was 12–82 years; 14.0% were male and 86.0% were female, and the affected side was the right in 50.0% and left in 48.0% of the patients, with bilateral disease observed in one patient. The thyroid gland was in a normal position in 66.0% of the patients, of which 9% were hypoplastic. In 34.0% of patients, the thyroid gland was not present at the correct location (Table 1). Preoperative definitive diagnosis was difficult, and although FNAC was performed in 70.4% of patients, the positive diagnosis rate was not high (60.0%). Although imaging studies, such as CT or ultrasound echography, are often indicative of an ectopic thyroid gland, it is not easy to make a preoperative diagnosis of an ectopic thyroid gland when a normal thyroid gland is present.

**Table 1:** Patient characteristics

Characteristics	(*N*=51)
Median age, years(range)	44(12-81)
Gender	male 8
	female 43
Affected side	right 26
	left 24
	both 1
Fine needle aspiration cytology	diagnosable 18
	unable to diagnosis 13
	not performed 14
	unknown 6
Pathology	thyroid tissue 28
	adenomatous goiter 12
	follicular adenoma 7
	no pathology 4
Normal thyroid gland	present 34 (hypoplastic 3)
	not present 17
Treatment	resection 39
	internal irradiation 1
	observation 8
	unknown 3
Thyroid replacement therapy	Yes 18 (total thyroidectomy 9)
	previously treated 2
	No 25
	unknown 6

In the present case, the mass had a high density on plain CT tomography, which was comparable to the density of the thyroid gland and reflected the iodine content of the tumors. However, because our patient had a normal thyroid gland and cytology showed only findings suggestive of cystic fluid and no cells of thyroid origin, we considered the findings to reflect an intracystic hemorrhage, and therefore, did not consider an ectopic thyroid gland as a differential diagnosis. Although generalization is difficult because masses often show cystic changes rather than uniform density, an ectopic thyroid should be included in the differential diagnosis when the tumor shows a density similar to that of the thyroid gland on plain CT images. If an ectopic thyroid is suspected preoperatively, thyroid scintigraphy can be useful for the diagnosis. However, this technique is not routinely performed for masses in the lateral neck. As the patient in the present case had a normal thyroid gland, an ectopic thyroid was not suspected, and scintigraphy was not performed. Differential diseases for a mass with cystic changes in the lateral neck region, as in the present case, include lateral cervical cysts, neurogenic tumors, and cystic cervical lymph node metastases. The mass was in contact with the submandibular gland, and a tumor of submandibular gland origin was therefore considered. Our case was atypical of a lateral cervical cyst, as there was a substantial component inside the cyst. Thyroid and oropharyngeal cancers are the most common diseases that can cause cystic cervical lymph nodes; however, in this case, there was no evidence of malignancy in the thyroid gland or oropharynx. Tumor resection was therefore performed to confirm the diagnosis.

If an ectopic thyroid is diagnosed preoperatively, follow-up is generally the treatment of choice. However, resection is often performed if the tumor grows, but it is preferable that the diagnosis be made preoperatively because ectopic tissue may be the only functional gland in 70% of cases [5]. Preoperative thyroid function tests should be performed and the possibility of postoperative hormone replacement therapy should be explained. Review of the past literature revealed that 80.9% of patients had undergone total resection of the mass; 19.1% of cases were diagnosed as ectopic thyroid by biopsy, cytology, or scintigraphy; and surgery was not performed. With the exception of two patients who had previously received hormone replacement therapy, 41.9% of the patients required thyroid hormone replacement after mass excision, and 50.0% of the cases in which replacement was initiated had undergone a previous or simultaneous total thyroidectomy. In contrast, in the remaining 50.0%, hypofunction became apparent only after mass removal, suggesting that the ectopic thyroid gland was the only functional gland. In the present case, no postoperative hypothyroidism was observed, suggesting that the normally positioned thyroid gland retained its a hormonal function. In previously-reported cases, the pathologic diagnosis of ectopic thyroid was thyroid tissue or hyperplasia in 60.9% and adenomatous goiter in 26.1% of cases. Follicular adenomas were found in 13.0% of the cases. There have been no reports of follicular adenomas with papillary architecture. Follicular adenomas have been described to have papillary structures in approximately 3% of cases. Follicular adenoma with papillary architecture was classified as benign neoplasms, independent of follicular adenoma in the fifth edition of the World Health Organization classification of endocrine tumors in 2022 [51]. In the previous edition, it was referred to as hyperfunctioning adenoma or follicular adenoma with papillary hyperplasia and were classified within category of follicular adenoma. This lesion is benign, and it is clinically important to differentiate it from other malignant tumors, particularly papillary carcinomas. Despite its papillary structure, this tumor can be distinguished from papillary carcinoma by the absence of nuclear characteristics, such as chromatin clearing, and by the presence irregularities in the nuclear membrane, such as grooves, folds, and pseudoinclusions, and enlargement. It is also distinguished from follicular carcinoma by the presence of a completely surrounding fibrous capsule and the absence of extracapsular or vascular invasion [52]. This is the first report of a follicular adenoma with papillary architecture that originated from an ectopic thyroid gland. When ectopic thyroid tumor is suspected, this disease should be included in the differential diagnosis.

## Conclusion

We report the first case of a follicular adenoma with papillary architecture originating from an ectopic thyroid gland. This case shows that although ectopic thyroid is very rarely found as a solitary tumor in the submandibular region, when papillary structures are seen on histopathology, this disease should be included in the differential diagnosis.

## Data Availability

No datasets were generated or analysed during the current study.

## Abbreviations

CT: computed tomography
FNAC: fine-needle aspiration cytology
